# Seroprevalence of visceral leishmaniasis among pregnant women in Jahrom city in Fars province, southern Iran

**DOI:** 10.1016/j.parepi.2024.e00349

**Published:** 2024-04-16

**Authors:** Najmeh Sadeghi, Mehdi Mohebali, Zahra Kakooei, Abazar Roustazadeh, Hamed Mir, Amir Abdoli, Kavous Solhjoo, Manoochehr Shabani, Enayatollah Shadmand, Ali Taghipour

**Affiliations:** a Zoonoses Research Center, Jahrom University of Medical Sciences, Jahrom, Iran; b Department of Parasitology and Mycology, School of Public Health, Tehran University of Medical Sciences, Tehran, Iran; c Center for Research of Endemic Parasites of Iran (CREPI), Tehran University of Medical Sciences, Tehran, Iran; d Department of Biochemistry and Nutrition, Jahrom University of Medical Sciences, Jahrom, Iran; e Department of Advanced Medical Sciences and Technologies, Jahrom University of Medical Sciences, Jahrom, Iran; f Department of Medical Parasitology and Mycology, School of Medicine, Jahrom University of Medical Sciences, Jahrom, Iran

**Keywords:** Iran, Pregnant women, Seroprevalence, Visceral leishmaniasis, Direct agglutination test

## Abstract

**Background:**

Visceral leishmaniasis (VL) is a public health issue in endemic countries with poor sanitation facilities. In this study, the seroprevalence rate and associated risk factors of VL were investigated during September 2020 to February 2021 in pregnant women referred to Ostad Mottahari and Peymanieh hospitals in Jahrom county, Fars province, southern Iran.

**Material and methods:**

A total of 220 serum samples of pregnant women were assessed for the presence of Anti*-Leishmania infantum* antibodies by direct agglutination antigen (DAT). The associated risk factors were obtained using questionnaires.

**Results:**

The overall seroprevalence of VL in pregnant women was 12.72% (28/220). Considering the antibody titer, titer 1:1600 was detected in 23 samples, titer 1:3200 in 4 samples, and titer 1:6400 in one sample. All 5 women with titer >3200 had mild fever. As such, there was a statistically significant difference regarding the age (≥39 years old with *p-*value: 0.01).

**Conclusions:**

We recommend an appropriate health education program for pregnant women and serological screening of VL before pregnancy in endemic cities. Moreover, we believed a need for more epidemiological studies for better understand the status of VL in pregnant women.

## 1 Background

*Leishmania donovani* and *Leishmania infantum/L.chagasi* cause visceral leishmaniasis (VL), also known as kala-azar (Murray et al., 2005). The female phlebotomine sandfly as vectors and canines as reservoir hosts are involved in both zoonotic and anthroponotic cycles of VL transmission (Murray et al., 2005). According to World Health Organization (WHO) reports, It is estimated that 50,000 to 90,000 new cases of VL occur worldwide annually (https://www.who.int/news-room/fact-sheets/detail/leishmaniasis). VL is endemic in tropical, subtropical and Mediterranean regions (Scarpini et al., 2022). About 90% of VL occur in poor rural and suburban areas of Bangladesh, India, Nepal, Sudan, and Brazil (Chappuis et al., 2007; Desjeux, 2004; Murray et al., 2005; Scarpini et al., 2022). Human and canine VL is endemic in several regions of Iran, including Fars, Northwest Azerbaijan, Ardabil of East Azerbaijan, Bushehr, North Khorasan, Chaharmahal & Bakhtiari, and Lorestan provinces (Mohebali, 2013; Mohebali et al., 2011; Sarkari et al., 2012). It has also been sporadically reported in other states in the country (Mohebali, 2013; Mohebali et al., 2011).The clinical manifestations of VL are chronic fever, cachexia, weakness, hepatosplenomegaly, small volume lymphadenopathy, blood cell reduction, paleness, or even pancytopenia and if untreated, death is as a complication of these parasites (Chappuis et al., 2007).

Weakening of the immune system plays a pivotal role for susceptibility against VL, while VL is a life-threatening infectious disease among immunocompromised patients, such as HIV/AIDS patients (Lindoso et al., 2018; Okwor and Uzonna, 2013). The immune system during pregnancy is altered in order to maintain the placenta and fetus, but this alteration can increase risk of congenital infections (Mor and Cardenas, 2010). Information about VL during pregnancy is limited, but it is known to be life-threatening to the mother (Berger et al., 2017; Figueiro-Filho et al., 2004). VL in pregnancy is rare and deserves special attention because very little information is known regarding the occurrence of VL during pregnancy and also textbooks of infectious diseases do not include this particular group (Caldas et al., 2003; Figueiro-Filho et al., 2004; Figueiró-Filho et al., 2008; Gradoni et al., 1994; Pagliano et al., 2005; Panagopoulos et al., 2017). However, no information is available regarding the status of VL among pregnant women in Iran. This study aimed to analyze the seroprevalence of VL among pregnant women and assess the risk and demographic factors in this community.

## 2 Materials and methods

### 2.1 Study area and study population

**Fig. 1:**
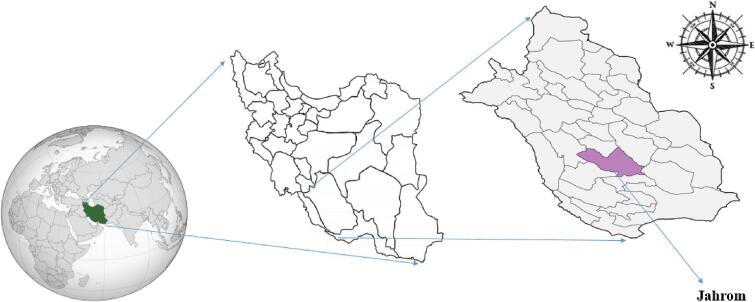
Geographic map of Jahrom City in Fars Province, Southern Iran.

This cross-sectional study was conducted from September 2020 to February 2021 in pregnant women referred to Ostad Mottahari and Peymanieh hospitals in Jahrom city, a city in southern Iran in Fars Province (Fig. 1) (Roustazadeh et al., 2021).

### 2.2 Sampling strategy

For this purpose, 220 confirmed pregnancies of women by clinicians and tests was considered as the inclusion criterion, and the consumption of anti-parasitic drugs during the 3 months period prior to sampling was the exclusion criteria. At first, the study objectives and protocol were explained to the pregnant women, by obtaining a written consent. During the study, all pregnant women agreed to participate in this study and no pregnant women were missed. Then, a designed structured questionnaire was used to record potential risk factors including: age, education status, occupation, blood type, number of pregnancies, history of abortion, month of pregnancy, history of diabetes, consuming raw vegetables, and eating raw or undercooked meat. We collected blood samples after receiving consent forms and questionnaires. This study received ethical approval from the Ethics Committee of the Jahrom University of Medical Sciences (No. IR.JUMS.REC.1401.029).

### 2.3 Direct agglutination antigen (DAT)

The DAT antigen was obtained through a multi-step process that included the mass generation of promastigotes of the Iranian strain L. *infantum* (MCAN/IR/07/Moheb-gh (no. GenBank Access) FJ555210)) in RPMI-1640 medium (Biosera, South America) furthermore 10% calf serum (Biosera, South America), after parasite trypsinization, stained with Coomassie Brilliant Blue R-250 (Sigma, St. Louis, Missouri, USA) and settled with 1.2% formaldehyde. Next, the serum samples that had been collected were subject to DAT testing in accordance with a prior investigation conducted in the leishmaniasis reference laboratory in the School of Public Health, Tehran University of Sciences, Iran (Mohebali et al., 2006a; Mohebali et al., 2020). For DAT testing, serum were evaluated at 1/800 dilution for screening and, in case of positive reaction, titration was done to 1:102400 dilution. The experimental plates had 96 (8 × 12) V-shaped wells. For screening procedure, 8-well rows were considered for one sample, while in case of titration 12-well rows were regarded for one specimen. To prepare sera dilutions, 90 μl human sera diluting agent and 10 μl sera were added to the first well to obtain 1:10 dilution. Subsequently, 10 μl of this mixture was added to the second well and incorporated with 90 μl human sera diluting agent, to obtain 1:100 dilution. In other wells, 50 μl human sera diluting agent and 50 μl human sera were mixed together, in order to yield 1:200, 1:400, 1:800, 1:1600, 1:3200, 1:6400, 1:12800, 1:25600, 1:51200 and 1:102400 dilutions, respectively. After the addition of 50 μl DAT antigen to the specific well with 1/800 dilution in screening, the plate was incubated at ambient temperature for 13–18 h. Of note, positive and negative control sera were considered for each set of experiments. In comparisons with positive and negative controls, compact blue dots were interpreted as negative, while large diffuse blue mats were positive (Mohebali et al., 2006a; Yimam Ayene et al., 2021). The test results were independently examined by two individuals. Based on previous studies (Mohebali et al., 2006b; Mohebali et al., 2011), specific anti-*Leishmania* antibodies at a titer of ≥1:3200 in humans sera were considered as positive case.

### 2.4 Statistical analysis

Data analysis for this study was performed using the SPSS software version 24. Differences between categorical variables were calculated using Pearson's chi-square test. A *P*-value <0.05 was considered statistically significant.

## 3 Results

### 3.1 Participants

A total of 220 serum samples were collected from pregnant women, aged from (≥18 years old) with a mean age of 32.8 years. In terms of education, 55.45% (122/220) in Jahrom had a diploma. Among the participants, 132 pregnant women (60%) were housewives, whereas 17 pregnant women (7.72%) were employees. Moreover, history of previous abortions was reported in 11 pregnant women (5%). The majority of women (59.54%) had more than one pregnancy. Most of the participants had blood type O (54.54%) with Rh positive (97.72%). It should be noted that we considered contact with cats and dogs as risk factors, but none of the participants were in contact with these (Table 1). More sociodemographic characteristics and risk factors of VL seroprevalence among pregnant women from Jahrom are summarized in Table 1.

**Table 1:** Seroprevalence of visceral leishmaniasis among pregnant women referred to the Ostad Mottahari and Peymanieh hospitals in Jahrom city, according to risk factors (*n* = 220).

Variable	Samples	Positivity		*P*-value
	N	N	%	
**Age (years)**				
18–23	34	3	8.82	Ref
24–28	51	4	7.84	0.88
29–33	68	5	7.35	0.81
34–38	43	5	11.62	0.71
39 or more	24	11	45.83	0.01⁎
**Education status**				
Illiterate	5	1	20	Ref
Primary and secondary school	45	5	11.11	0.61
Diploma	122	16	13.11	0.70
College	48	6	12.5	0.68
**Occupation**				
Housewife	132	15	11.36	Ref
Self-employed	71	9	12.67	0.80
Employee	17	4	23.52	0.23
**Blood type**				
A	39	5	12.82	0.65
B	47	10	21.27	0.09
AB	14	1	7.14	0.75
O	120	12	10	Ref
**Rh**				
+	215	28	13.02	Ref
−	5	0	0	NA
**Previous abortions**				
Yes	11	1	9.09	0.74
No	209	27	12.91	Ref
**Pregnancy number**				
Once	89	10	11.23	0.62
More than once	131	18	13.74	Ref
**Month of pregnancy**				
First trimester	51	5	9.80	0.28
Second trimester	83	8	9.63	0.19
Third trimester	86	15	17.44	Ref
**History of diabetes**				
Yes	9	4	44.44	0.25
No	111	24	21.62	Ref
**Consuming raw vegetables**				
Yes	89	15	16.85	0.18
No	131	13	9.92	Ref
**Eating raw or undercooked meat**				
Yes	113	18	15.92	Ref
No	107	10	9.34	0.19
**Contact with cats and dogs**				
Yes	0	0	0	NA
No	220	28	12.72	Ref

NA: not applicable.

Ref: A reference group is a group to which an individual or another group is compared.

⁎ *P-*value <0.05 was considered statistically significant.

### 3.2 Seroprevalence and risk factors of VL

The overall seroprevalence of VL in pregnant women was 12.72% (28/220). Considering the antibody titer, titer 1600 was detected in 23 samples, titer 3200 in 4 samples, and titer 6400 in one sample. Only samples with titers of ≥3200 are considered positive for VL and require clinical examinations. If these pregnant women have specific symptoms (mild fever for more than two weeks, hepatosplenomegaly, and pancytopenia) during clinical examinations, they are considered positive for VL. All 5 women with titer >3200 had mild fever. As shown in Table 1, there was a statistically significant difference regarding the age (39 ≤ years old with *P-*value: 0.01). There was not a statistically significant association between seropositivity status with education status, occupation, blood type and Rh, previous history of abortions, pregnancy number, month of pregnancy, history of diabetes (Table 1).

## 4 Discussion

In the present study, DAT showed the overall seropositivity of 12.72% (28/220), of which, five samples had antibody titers >3200. While only samples with titers of 3200 and more in the DAT test are considered positive for VL, these patients were referred to the specialist physician for further clinical practice. The results of a meta-analysis demonstrated that the estimated pooled seroprevalence of VL was 2% (95% CI: 1–2%) among the general population of Iran (Rostamian et al., 2021). Comparison of these results with our analysis revealed that pregnant women could be a susceptible group for VL. Among the different serology tests, DAT was specific (72–100%) and sensitive (92–100%) for diagnosis of VL, especially in endemic regions of the world (Mohebali et al., 2006a; Mohebali et al., 2020).

Most studies of VL in pregnant women have been reported as case report or case series (Caldas et al., 2003; Cunha et al., 2019; Figueiro-Filho et al., 2004; Figueiró-Filho et al., 2008; Gradoni et al., 1994; Mueller et al., 2006; Pagliano et al., 2005; Panagopoulos et al., 2017; Silva et al., 2015). A systematic review of the literature revealed 17 cases of VL during pregnancy that untreated VL resulted in consequences on the fetus or congenital VL (Pagliano et al., 2005). Among these pregnant women, none had underlying diseases and fever was reported in all cases, weight loss in three and abdominal pain in two. Four women were in their first pregnancy, and one in her second. The onset of clinical symptoms varied between 1 and 12 weeks (median 9 weeks) before diagnosis (Pagliano et al., 2005). In our study, all 5 women with titer >3200 had mild fever.

Analysis of VL among pregnant and non-pregnant women in Sudan revealed that pregnant women with VL had more severe anemia and were more likely to need blood transfusion (OR 9.3; 95% CI 2.5–34.2) compared to non-pregnant women with VL (Pekelharing et al., 2020). It is suggested to consider the number of blood cells and blood disorders (such as anemia) in future studies. Adverse pregnancy outcomes, including premature delivery and miscarriage, were reported in 20% pregnant women with VL, and 50% where VL was diagnosed postpartum. Nevertheless, mortality was low (1.8%) and initial cure rates were high (96.5%) among pregnant women with VL (Pekelharing et al., 2020).

Mueller et al. performed a retrospective study in Sudan regarding treatment of VL among pregnant women (Mueller et al., 2006). In this regard, treatment with sodium stibogluconate, AmBisome plus sodium stibogluconate, and AmBisome alone were performed among 23, 4, and 12 women, respectively. It was observed, spontaneous abortions were occurred in 13 (57%) of the patients that were treated with sodium stibogluconate monotherapy. Except for one patient that was treated with sodium stibogluconate, other patients were cured and discharged in good clinical condition (Mueller et al., 2006). Treatment with antimoniate (Utili et al., 1995) and amphotericin B (Topno et al., 2008) were also used in treatment of most pregnant women with VL (see the systematic review (Dahal et al., 2021)). In the present study, the type of treatment for VL in pregnant women was not followed up, but it is necessary for clinicians to consider the best drug treatment in infected pregnant women.

Congenital transmission of VL was also reported in some cases. In this regard, Zinchuk and Nadraga (Zinchuk and Nadraga, 2010) reported VL in an 8-month-old boy whose mother had been treated for VL at 28–32 weeks of gestation and her infant was delivered at 38 weeks of gestation by elective caesarean section. Another report was from a 16-month-old German boy with a 4-week history of weakness, fatigue, intermittent fever, difficulty sleeping, and decreased appetite. He was successfully treated with sodium stibogluconate and his symptoms disappeared after treatment (Meinecke et al., 1999). Two cases of congenital transmission of VL were also reported from 23- and 24-years pregnant women in Brazil (Mescouto-Borges et al., 2013). In our study, the highest positive antibody titer was observed in the third trimester of pregnancy, so it is suggested to conduct cohort studies considering the month of pregnancy and the condition of the child after birth.

In our study, age (≥39 years old) was significantly associated with the seroprevalence of VL. In this regard, it is possible that the chance of exposing *Leishmania* infection increases with age, as a result, a higher titer was seen in older pregnant women (Chapman et al., 2018).

To the best of our knowledge, this is the first study that reports VL in pregnant women in Iran. Analysis of 260 cases with VL in Fars Province, Southern Iran, revealed that the mean age of patients was 3.5 years and the main clinical symptoms were fever (96.2%), abdominal protrusion (71.9%), and hepatosplenomegaly (68.8%). It is suggested to consider clinical symptoms and signs in future studies. Delay in the detection and treatment of VL can lead to high mortality and morbidity in patients. Hence, screening of VL should be focused on susceptible groups, including pregnant women. Asymptomatic stray dogs act as main reservoirs of VL in the endemic areas of Iran (Shokri et al., 2017). Hence, control of these animals could help for prevention of VL to humans.

## 5 Conclusion

Our study revealed the overall seropositivity rates of VL was 12.72% among pregnant women in Jahrom county, which is more than the overall seroprevalence of VL (2%, 95% CI: 1–2%) among the general population of Iran (Rostamian et al., 2021). Hence, we recommend an appropriate health education program for pregnant women and serological screening of VL before pregnancy in endemic cities. However, more studies are needed regarding establishment of a surveillance system for early detection of VL particularly in the endemic areas of VL.

## Author contributions

AT and NS designed the study. HM and AR collected the samples and prepared them for laboratory processing. MM and ZK performed laboratory experiments and analyzed the data. AT and NS wrote the draft of the manuscript. AT, ES and MS supervised the project and finalized the manuscript. All the authors commented on the manuscript and approved the final version of the paper.

## Funding

This research has been supported by the Jahrom University of Medical Sciences, Iran.

## Availability of data and materials

All data during study are included in this manuscript.

## Ethics approval and consent to participate

This study received ethical approval from the Ethics Committee of the Jahrom University of Medical Sciences (No. IR.JUMS.REC.1401.029).

## Consent for publication

Not applicable.

## Declaration of competing interest

The authors declare no conflict of interest.
